# Differential Deployment of REST and CoREST Promotes Glial Subtype Specification and Oligodendrocyte Lineage Maturation

**DOI:** 10.1371/journal.pone.0007665

**Published:** 2009-11-03

**Authors:** Joseph J. Abrajano, Irfan A. Qureshi, Solen Gokhan, Deyou Zheng, Aviv Bergman, Mark F. Mehler

**Affiliations:** 1 Institute for Brain Disorders and Neural Regeneration, Albert Einstein College of Medicine, Bronx, New York, United States of America; 2 Department of Neurology, Albert Einstein College of Medicine, Bronx, New York, United States of America; 3 Department of Neuroscience, Albert Einstein College of Medicine, Bronx, New York, United States of America; 4 Department of Psychiatry and Behavioral Sciences, Albert Einstein College of Medicine, Bronx, New York, United States of America; 5 Department of Pathology, Albert Einstein College of Medicine, Bronx, New York, United States of America; 6 Department of Genetics, Albert Einstein College of Medicine, Bronx, New York, United States of America; 7 Department of Systems and Computational Biology, Albert Einstein College of Medicine, Bronx, New York, United States of America; 8 Einstein Cancer Center, Albert Einstein College of Medicine, Bronx, New York, United States of America; 9 Rose F. Kennedy Center for Research on Intellectual and Developmental Disabilities, Albert Einstein College of Medicine, Bronx, New York, United States of America; Universidade Federal do Rio de Janeiro (UFRJ), Instituto de Biofísica da UFRJ, Brazil

## Abstract

**Background:**

The repressor element-1 (RE1) silencing transcription factor/neuron-restrictive silencer factor (REST/NRSF) is a master transcriptional regulator that binds to numerous genomic RE1 sites where it acts as a molecular scaffold for dynamic recruitment of modulatory and epigenetic cofactors, including corepressor for element-1-silencing transcription factor (CoREST). CoREST also acts as a hub for various cofactors that play important roles in epigenetic remodeling and transcriptional regulation. While REST can recruit CoREST to its macromolecular complex, CoREST complexes also function at genomic sites independently of REST. REST and CoREST perform a broad array of context-specific functions, which include repression of neuronal differentiation genes in neural stem cells (NSCs) and other non-neuronal cells as well as promotion of neurogenesis. Despite their involvement in multiple aspects of neuronal development, REST and CoREST are not believed to have any direct modulatory roles in glial cell maturation.

**Methodology/Principal Findings:**

We challenged this view by performing the first study of REST and CoREST in NSC-mediated glial lineage specification and differentiation. Utilizing ChIP on chip (ChIP-chip) assays, we identified distinct but overlapping developmental stage-specific profiles for REST and CoREST target genes during astrocyte (AS) and oligodendrocyte (OL) lineage specification and OL lineage maturation and myelination, including many genes not previously implicated in glial cell biology or linked to REST and CoREST regulation. Amongst these factors are those implicated in macroglial (AS and OL) cell identity, maturation, and maintenance, such as members of key developmental signaling pathways and combinatorial transcription factor codes.

**Conclusions/Significance:**

Our results imply that REST and CoREST modulate not only neuronal but also glial lineage elaboration. These factors may therefore mediate critical developmental processes including the coupling of neurogenesis and gliogenesis and neuronal-glial interactions that underlie synaptic and neural network plasticity and homeostasis in health and in specific neurological disease states.

## Introduction

The repressor element-1 silencing transcription factor/neuron-restrictive silencer factor (REST/NRSF) is a master transcriptional and post-transcriptional regulator [1] that modulates distinct sets of protein-coding and non-coding genes in specific cell types, such as embryonic stem cells (ESCs) and neural stem cells (NSCs) [2], and has a broad array of context-specific functions including the regulation of embryonic development [3], neurogenesis [4], [5], synaptic plasticity [4], neurosecretory mechanisms [6], and extracellular matrix composition [7]. Aberrant REST expression and function are implicated in diverse disorders including cancer [8], neurodegeneration [9] and neurodevelopmental diseases [10]. REST was initially believed to repress expression of genomic repressor element-1 (RE1) motif containing neuronal differentiation genes in NSCs and in non-neuronal cells by recruiting chromatin remodeling enzymes and other regulatory cofactors to its N- and C-terminal binding domains, including the corepressor for element-1-silencing transcription factor (CoREST) to its C-terminus [11], [12], [13], to form a modular macromolecular complex. REST is now believed to have an increasing spectrum of developmental stage- and cell type-specific functions, including gene activation, repression, and long-term gene silencing, that are modulated by factors such as the levels of REST protein expression, the affinity of the REST complex for specific genomic loci, and the presence of regulatory cofactors (e.g., modulatory double-stranded ncRNAs and distinct isoforms of REST) [11], [14], [15], [16].

Like REST, CoREST also regulates neuronal gene expression by acting as a scaffold for the recruitment of various epigenetic factors that play roles in chromatin remodeling, including MeCP2, HDAC1/2, LSD1, BHC80, and BRAF35 [17], [18]. Distinct CoREST complexes can bind to REST or function independently in order to modulate target gene expression [4], [19]. For example, one study demonstrated that for a subset of neuronal genes, designated class I, absence of the REST complex results in maximal levels of gene expression, whereas for class II neuronal genes, absence of the REST complex only results in submaximal levels of gene expression due to repressive effects from a separate CoREST complex bound to distinct sites on the promoters of these genes [4].

Although various studies have identified genes that are targets for REST in ESCs, NSCs, and other cell types [20], [21], [22], [23], [24], a detailed understanding of the roles played by REST and CoREST in governing developmental gene expression programs is still emerging. In this study, we characterize developmental stage-specific profiles for REST and CoREST target genes in glial cells, including thousands of genes not previously described as targets for REST regulation. These include factors known to be involved in the acquisition and maintenance of macroglial (astrocyte [AS] and oligodendrocyte [OL]) cell identity and functions as well as those that have not been previously associated with glial cell biology. These findings now implicate REST and CoREST in a previously unrecognized role—regulation of glial lineage elaboration, including AS and OL specification and progressive stages of OL lineage maturation.

Our observations suggest that REST and CoREST are key nodes in the epigenetic regulatory circuitry governing both neuronal and glial gene expression and may also be responsible for coordinating AS functions, such as controlling trophic neural microenvironments and modulating synaptic plasticity, and OL functions, such as mediating neuronal-glial interactions and axonal myelination.

## Results

We examined the molecular mechanisms underlying glial lineage elaboration by assessing REST and CoREST protein expression and profiling of REST and CoREST target genes using ChIP-chip analysis in a developmental paradigm comprised of both immature and mature glial cell types. We identified profiles for REST and CoREST target genes that are unique to specific cell types as well as those found in combinations throughout critical developmental transitions associated with glial lineage specification and progressive stages of OL lineage maturation. We examined these transitions to better understand the specific ways in which REST and CoREST modulate glial developmental states and gene expression programs. Specifically, we studied the following critical developmental transitions: specification of astrocytes [neural stem cells → astrocytes (NSCs → ASs)], specification of OL precursors [bipotent progenitor cells → OL precursors (N/OPs → OLpres)], and progressive stages of OL lineage maturation including myelination [OL precursors → OL progenitors (OLpres → OL pros), OL progenitors → post-mitotic OLs (OLpros → pmOLs), and post-mitotic OLs → mature, myelin expressing OLs (pmOLs → myOLs)]. In addition, we correlated REST and CoREST promoter occupancy with corresponding gene expression profiles to interrogate their potential regulatory roles in macroglial lineage elaboration.

### REST and CoREST Expression and Subcellular Localization

**Figure 1:**
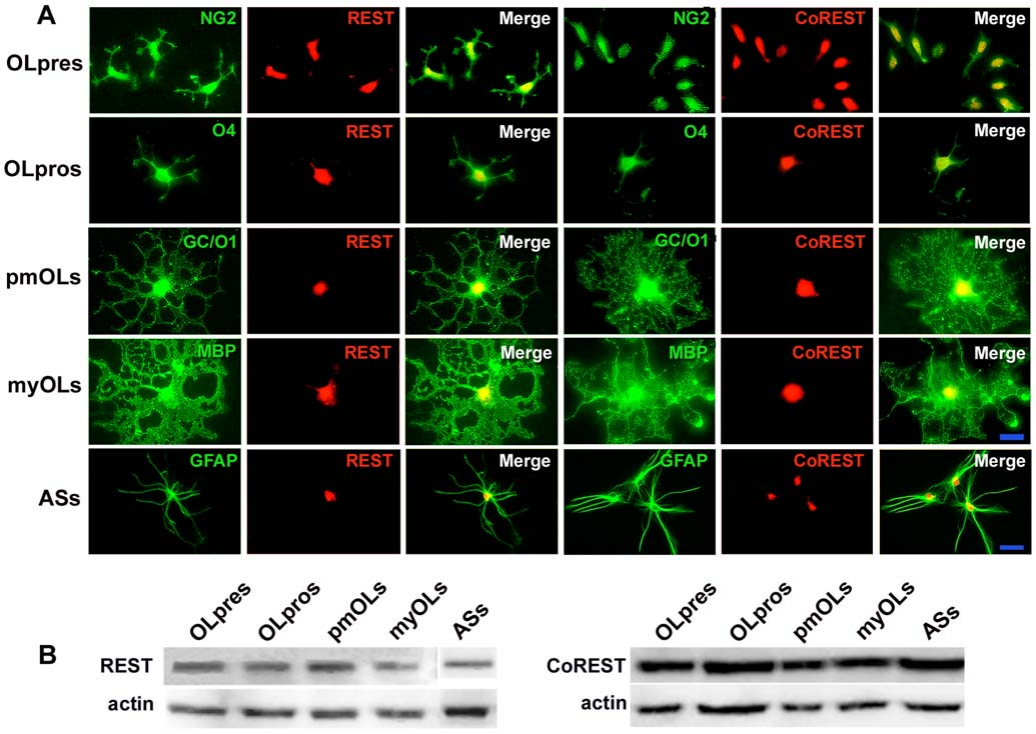
Expression and subcellular localization of REST and CoREST in glial developmental species. (A) Immunofluorescence microscopy of REST and CoREST (TRITC) expression profiles in glial developmental cell types. REST and CoREST are expressed in the nucleus and cytoplasm of all glial cell types, including astrocytes (AS), oligodendrocyte (OL) precursors (OLpre), OL progenitors (OLpro), post-mitotic OLs (pmOL), and mature, myelin expressing OLs (myOLs). Antibodies to specific OL and AS developmental markers (FITC) were used to identify specific stages of glial cell maturation. Scale bars = 50 µm (OLpres-myOLs) and 10 µm (ASs), respectively. (B) Western blot analysis of REST and CoREST expression in glial developmental species. REST and CoREST are ubiquitously expressed in all cell types examined in our developmental paradigm.

We measured REST and CoREST expression and subcellular localization throughout glial lineage elaboration by performing immunofluorescence microscopy and Western blot analysis (Figure 1). We found that both proteins are ubiquitously expressed in the nucleus and cytoplasm of all cell types examined in our developmental paradigm. Together, these observations suggest that REST and CoREST are present in all glial cell types and therefore have the potential to regulate gene expression profiles during glial subtype specification and progressive stages of OL lineage maturation.

### Genome-Wide Promoter Occupancy Profiles for REST and CoREST

**Figure 2:**
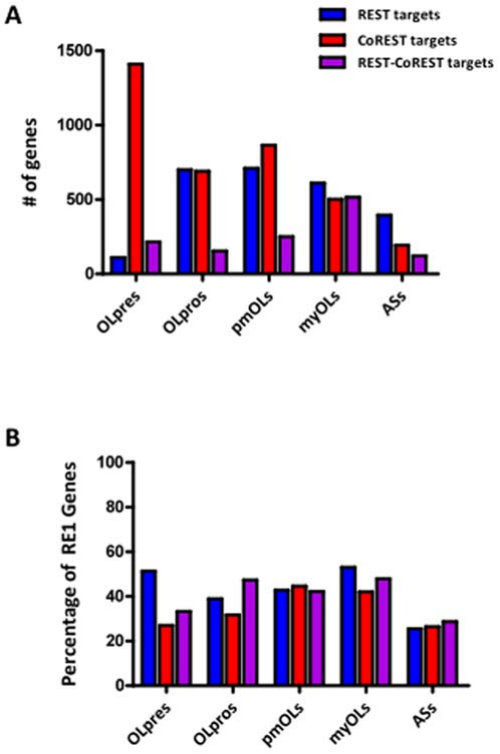
Profiles of REST and CoREST target genes in macroglial developmental cell types. The number of exclusive REST, exclusive CoREST, and REST and CoREST (REST-CoREST) target genes uniquely present in individual glial cell types as identified through chromatin immunoprecipitation on promoter chip (ChIP-chip) experiments. We identified a total of 3,178 REST target genes and 4,060 CoREST target genes. Note the presence of a disproportionately high number of CoREST target genes in OLpres. (B) The percentages of REST, CoREST, or REST-CoREST target genes present in individual glial developmental cell types that contain previously characterized repressor element-1 (RE1) motifs [20], [22].

While REST and CoREST expression and subcellular localization remain relatively constant, we found that there is significant variation in the number of promoter sites they occupy in each of the different glial developmental cell types (Figure 2A). We found a total of 3,178 REST and 4,060 CoREST target genes across all glial cell types examined (Table S1). Amongst these genes, we found that 1,535 are “exclusive” targets of REST; 2,417 are exclusive targets of CoREST; and 1,643 are targets of both REST and CoREST (REST-CoREST). We designated these genes as “unique” targets if they were bound solely within one of the glial cell types examined. For ASs, we identified 287 unique and exclusive REST target genes; 40 unique and exclusive CoREST target genes; and 10 unique REST-CoREST target genes. For OLpres, OLpros, pmOLs, and myOLs, we identified 127, 398, 465, and 502 unique and exclusive REST target genes; and we found 767, 312, 346, and 305 unique and exclusive CoREST target genes. For OLpres, OLpros, pmOLs, and myOLs we further identified 60, 20, 43, and 131 unique REST-CoREST target genes. Interestingly, these observations demonstrate a progressive increase in the number of unique and exclusive REST target genes during OL lineage maturation, suggesting that REST may acquire more diverse roles as OLs undergo progressive lineage maturation, cell cycle exit, and myelination. In contrast, CoREST binds to a disproportionately large number of unique and exclusive target genes in OLpres, implying that it may have a preferential role in OL lineage specification. These striking observations reveal that REST and CoREST have distinct profiles of target genes in each glial subtype and suggest that they perform largely non-overlapping developmental stage-specific regulatory functions during glial lineage elaboration.

Further, we compared glial target genes with a previously characterized set of RE1-containing genes (Figure 2B) [20], [22]. We observed that the percentages of RE1-containing REST, CoREST, and REST-CoREST target genes range from 25.6% to 53.0% across OL developmental lineage species. Alternatively, for the AS lineage, we found a more consistent percentage of RE1-containing target genes at the lower end of this range, with REST, CoREST, and REST-CoREST targets having 25.6%, 26.4%, and 28.7%, respectively. These results demonstrate that, in glial cells, REST and CoREST primarily target genes that have not previously been characterized as containing RE1 motifs. These cumulative observations support the conclusion that differential REST and CoREST DNA binding motifs may encode lineage-specific functional information [25].

**Figure 3:**
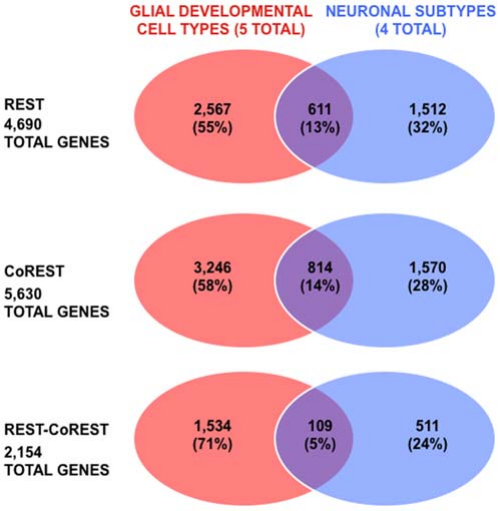
Comparative analysis of REST and CoREST target gene profiles in glial and neuronal subtypes. The distinct and overlapping profiles of REST, CoREST and REST-CoREST target genes present throughout glial lineage elaboration and in neuronal subtypes [cholinergic neurons (CHOLNs), medium spiny projection neurons (MSNs), GABAergic neurons (GABANs), glutamatergic neurons (GLUTNs)]. These observations are highly statistically significant with corresponding p-values<0.0001 (i.e., cumulative probability calculated using hypergeometric testing) for the number of overlapping glial and neuronal genes in each comparison.

To begin to characterize the distinct roles of REST and CoREST in modulating glial gene expression programs, we compared profiles of REST and CoREST target genes from all glial cell types to those from a corresponding study we performed in neuronal subtypes (Figure 3). Unexpectedly, we found that neuronal and glial REST and CoREST target gene profiles are quite distinct. We observed that only 13% of REST target genes in glia are also REST targets in neurons, 14% of CoREST target genes in glia are also CoREST targets in neurons, and 5% of REST-CoREST target genes in glia are also REST-CoREST targets in neurons. These observations are highly statistically significant with corresponding p-values<0.0001 (i.e., cumulative probability calculated using hypergeometric testing) for the number of overlapping glial and neuronal genes in each comparison. These findings show that a relatively small number of REST and CoREST target genes are common to both neuronal and glial lineages and strongly suggest that REST and CoREST regulate distinct sets of genes and developmental events during neuronal and glial lineage elaboration.

### REST and CoREST Target Gene Profiles during Seminal Developmental Transitions in Glial Lineage Elaboration

**Figure 4:**
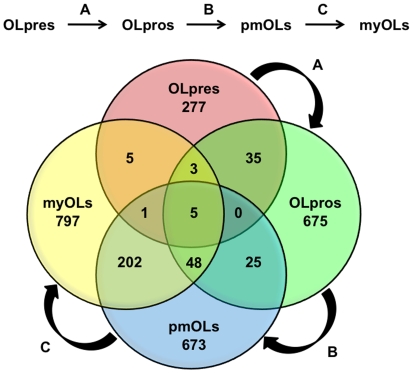
Comparative profiles of REST target genes present during seminal stages of oligodendrocyte lineage elaboration. We examined the distinct and overlapping profiles of REST target genes present in OL lineage species during progressive stages of OL lineage maturation (OLpre → OLpro → pmOL → myOL).

**Figure 5:**
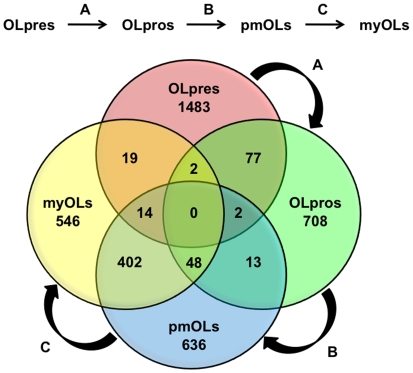
Comparative profiles of CoREST target genes present during seminal stages of oligodendrocyte lineage elaboration. We examined the distinct and overlapping profiles of CoREST target genes present in OL lineage species during progressive stages of OL lineage maturation (OLpre → OLpro → pmOL → myOL).

In order to better understand the potential maturational and maintenance functions of REST and CoREST, we identified subsets of genes that were targeted throughout seminal developmental transitions in glial lineage elaboration (Figures 4 and 5). We found that the number of REST target genes shared between OL lineage species increases during progressive maturation. The transitions between OLpres → OLpros, OLpros → pmOLs, and pmOLs → myOLs included cell types that shared 43, 78, and 256 target genes, respectively. For CoREST, we observed that the transitions between OLpres → OLpros, OLpros → pmOLs, and pmOLs → myOLs included cell types that shared 81, 63, and 464 target genes, respectively. In parallel with the findings for REST, the final OL lineage transition (pmOLs → myOLs) included cell types that shared the largest number of CoREST target genes. The degree of overlap between profiles of REST and CoREST targets during these OL developmental transitions, particularly the final transition, suggests that highly complex and integrated transcriptional regulatory mechanisms involving REST and CoREST are required to orchestrate the expression of genes involved in OL maturation and maintenance. These profiles imply that shared genes may be regulated similarly in different developmental stages, where they may either be dynamically activated, repressed, or primed for subsequent actions. Further, the numbers of shared targets during early developmental transitions are significantly less than in the final transition suggesting that regulation of largely distinct subsets of genes may be important for earlier transitions while more common regulatory modules are responsible for terminal differentiation and maturation.

We also identified specific genes targeted throughout all developmental stages within the OL lineage (e.g, OLpres, OLpros, pmOLs, and myOLs). We found five genes that are targeted by REST, including genes encoding membrane proteins (*Syt4* and *Gm691*), a neuroendocrine secretory protein (*Scg3*), a G-protein coupled receptor (*Gpr158*), and a solute carrier (*Slc12A5*). In contrast, we found that CoREST did not target any genes during all stages of OL lineage elaboration. Together, these findings imply that differential deployment of REST and CoREST may be important for mediating seminal developmental processes throughout progressive stages of OL lineage maturation.

### Pathway Analysis of Composite Profiles of REST and CoREST Target Genes

To compare the putative functional roles of REST and CoREST, we analyzed composite profiles of REST and CoREST target genes from all glial cell types using Ingenuity Pathways Analysis (Table S2). Exclusive REST target genes and exclusive CoREST target genes were selectively enriched for distinct pathways highlighting potential differences in the roles played by REST and CoREST in regulating developmental processes important for glial lineage specification and maturation. Specifically, for exclusive REST target genes, these pathways included TGF-β signaling, LPS-stimulated MAPK signaling, estrogen receptor signaling, and VEGF signaling, whereas for exclusive CoREST target genes these pathways included actin cytoskeleton signaling, IGF-1 signaling, and PDGF signaling. Moreover, we also identified commonly enriched pathways for exclusive REST target genes and exclusive CoREST target genes, suggesting that REST and CoREST modulate the same developmental processes through different subsets of genes. These pathways included: ephrin receptor signaling, integrin signaling, and axonal guidance signaling. In addition, for REST-CoREST target genes, we identified enriched pathways for Wnt/β-catenin signaling, glutamate receptor signaling, and the ole of BRCA1 in DNA damage response.

### Pathway Analysis of REST and CoREST Target Genes in Individual Glial Cell Types

To further examine the potentially distinct and overlapping roles for REST and CoREST within individual glial cell types, we examined REST and CoREST target genes in ASs and throughout progressive stages of OL lineage maturation (Table S3). For REST targets, we identified enriched pathways involved in a diverse set of biological functions. For example, in ASs, these pathways included the tyrosine metabolism pathway. In OLpres, these pathways included the cytochrome P450 pathway. In pmOLs, these pathways included glucocorticoid receptor signaling, protein ubiquitination pathway, and EGF signaling. Finally, in myOLs, these pathways included death receptor signaling, apoptosis, Wnt/β-catenin signaling, GABA receptor signaling, and estrogen receptor signaling.

Similarly, we found that CoREST targets are also involved in a distinct and diverse array of biological pathways. In ASs, these pathways included riboflavin metabolism. In OLpres, these pathways included androgen and estrogen metabolism and the complement system. In OLpros, these pathways included thyroid hormone receptor/retinoid X receptor (TR/RXR) activation and p53 signaling. In pmOLs, these pathways included axonal guidance signaling and amyloid processing. Finally, in myOLs, these pathways included Wnt/β-catenin signaling, death receptor signaling, glutamate receptor signaling, neuregulin signaling, synaptic long-term potentiation, and GABA receptor signaling.

### REST and CoREST Target Genes Mediate Diverse Developmental Processes in Glial Lineage Species

#### Astroglial functions

We identified unique REST and CoREST target genes in ASs that modulate various functional properties of ASs (Table S4). For example, we identified target genes that encode enzymes involved in prostaglandin biosynthesis and in modulating AS activation in response to brain injuries, including Alox5 [26] and Ptgs2 [27], [28]. In addition, we also found target genes that are part of a recently described AS transcriptome database [29] such as Wsb1, a SHH regulated E3 ubiquitin ligase, which modulates cell proliferation, viability and stress responses [30] and may be involved in mediating the increased proliferative activity observed in activated ASs [31].

#### Oligodendrocyte specification and progressive maturation

In each progressive stage of OL lineage maturation, we uncovered a range of unique REST and CoREST target genes encoding factors with roles in shaping early OL development as well as in regulating the intrinsic OL developmental rheostat that controls the fidelity of cell cycle exit and terminal differentiation including myelinlation (Table S4) [32]. For example, in OLpres, we found target genes encoding Cntn1/F3, an immunoglobulin superfamily cell adhesion molecule critical for promoting OL maturation [33], [34], [35]; Tcf4, a high mobility group (HMG) family downstream effector of Wnt signaling and transcriptional inhibitor of OL maturation and myelination genes [36], [37], [38], [39]; and Mobp, an essential myelin protein [40]. In OLpros, we identified target genes encoding Sema3a, a guidance cue that directs OL migration [41]; CD9, a tetraspanin family member expressed in premyelinating OLs [42]; S100a10, an S100 family member involved in cell cycle progression and OL differentiation [43]; and LIFR, a cytokine receptor that promotes anti-apoptotic responses in OLs and modulates cuprizone-induced demyelination and myelin repair [44]. In pmOLs, we target genes that encode factors including Gap43, a gap junction protein, and Sept5, a septin cytoskelton protein, that are both involved in determining OL morphology; Ccndbp1, Hoxa2, Olig2, and Sox4, which are all responsible for transcriptional regulation of OL genes; and MBP, which determines myelination status. In myOLs, we identified genes that encode factors promoting OL differentiation and myelin gene expression. Specifically, Zfp488 is an OL-specific transcriptional co-regulator of Olig2 [45], and Myt1 is a transcription factor that mediates the transition between immature OL proliferation and terminal OL differentiation while inducing expression of myelin genes [46]. We also identified target genes that are highly expressed in myOLs (e.g., *Tmem10/Opalin*) [47], [48], where they may promote OL terminal differentiation and myelination.

In various combinations and permutations, REST and CoREST also target genes encoding factors with key roles in OL specification and progressive maturation, including basic helix loop helix (bHLH) (e.g., *Mash1/Ascl1, NeuroD4/Math3,* and *Id4*), high-mobility group (HMG) (e.g., *Sox2, Sox8,* and *Sox11*), and POU domain (e.g., *Pou3f2*) transcription factors (Table S4). Mash1 acts in a cell- and maturational stage-specific manner and is involved in the elaboration of bipotent neuronal-oligodendrocyte progenitors (N/OPs) and in promoting OL lineage specification, terminal maturation, and myelin gene expression [49], while NeuroD4/Math3 plays an even earlier role in neuronal versus glial fate determination [50]. We identified other target genes encoding factors with roles in OL lineage maturation [51], including Sox2, important in OL cell cycle progression [52]; Sox11, expressed in OLpres where it is thought to inhibit OL terminal differentiation [53]; and Pou3f2, important for promoting Sox11 developmental functions [54]. Interestingly, we also identified Sox8, another Sox protein with dual roles in OL lineage progression and myelination. Specifically, during early OL specification, Sox8 has an accessory role in progressive OL maturation by cooperating with Sox9 [55]. In contrast, during later OL myelination, Sox8 cooperates with Sox10 to induce the expression of myelin factors (e.g., MBP and PLP) [55], [56], [57]. Moreover, we identified target genes encoding other factors involved in progressive OL maturation, including: Kcnj10, an inward-rectifying potassium channel required for OL terminal differentiation and myelination [58]; Nog, a modulator of TGF-β signaling essential for the development of ramified OLs and myOLs [59]; Itgb1, an integrin required for mediating OL axonal contact during myelination [60]; Mpzl1, a protein similar to an integral peripheral myelin component [47], [61]; NF1, a membrane glycoprotein that modulates MBP gene transcription [62]; ID4, a factor that differentially regulates myelination by repressing MBP but inducing other myelin proteins, including PLP and MAG [63]; and MBP, a myelin protein critical for OL terminal differentiation and myelination [52], [64].

Integrating our ChIP-chip data with gene expression data, including our own (Table 1) and previous studies [47], extends and further suggests that REST and CoREST may be critical for promoting the timing and fidelity of OL lineage elaboration by sequentially modulating of expression of immature and mature OL lineage factors and regulating the expression of factors essential for myelin gene programs. These findings may have important implications for OL biology and for our understanding of the pathogenesis of a spectrum of dysmyelinating and demyelinating diseases as well as processes that may be recapitulated during the process of remyelination following injury or disease states [65], [66]. These observations are also consistent with the hypothesis that the REST complex may prime the local chromatin environment for hierarchical developmental gene expression similar to other epigenetic modifications [25].

**Table 1:** Comparative analysis of REST and CoREST promoter occupancy and corresponding gene expression profiles during glial developmental transitions.

		REST	CoREST
*A*	*B*	+/−	+/−
**NSC**	**AS**		
No	Yes	184/48	135/10
Yes	No	23/94	90/270
**N/OP**	**OLpre**		
No	Yes	130/32	299/167
Yes	No	10/22	19/26
**OLpre**	**OLpro**		
No	Yes	126/49	95/26
Yes	No	30/13	166/39
**OLpro**	**pmOL**		
No	Yes	41/127	73/169
Yes	No	52/85	28/61
**pmOL**	**myOL**		
No	Yes	197/83	109/54
Yes	No	124/60	111/48

We examined REST and CoREST target gene expression profiles during the specification of ASs (NSCs→ASs) and OLpres (N/OPs→OLpres) and during progressive stages of OL lineage maturation (OLpres→OLpros, OLpros→ pmOLs, and pmOLs→myOLs). The absence or presence of REST and CoREST promoter occupancy for target genes within each cell type are indicated by no and yes. Each pair of numbers represents genes up regulated and down regulated (+/−), respectively, during the transition from the proximal progenitor (column A) to its immediate progeny (column B).

#### Epigenetic regulation

Epigenetic regulation is now emerging as an important cellular mechanism for mediating the interplay between extrinsic and intrinsic signals, such as morphogenic signaling pathways and transcription factor codes, to determine cell type specific gene expression profiles [67], [68]. In fact, REST and CoREST recruit a diverse group of epigenetic factors to their regulatory complexes where they play roles in cell type specific transcriptional regulation and in cell fate determination [11], [15]. Intriguingly, we found that REST and CoREST target a large cohort of genes encoding epigenetic factors (Table S5) including DNA methylation factors, such as DNA methyltransferases (e.g., *Dnmt1*) and methyl-CpG binding domain (MBD) proteins (e.g., *Mbd2*, *Mbd3*, and *Mbd6*); members of the SWI/SNF family of chromatin remodeling enzymes (e.g., *Smarcd2, Smarcc1, Smarcb1, Smarcal1, Smarcad1, Smarca3, Smarca2,* and *Smarca1*); histone modifying enzymes, such as histone deacetylases (e.g., *Hdac6* and *Hdac7a*), histone demethylases (e.g., *Jmjd1a, Kdm5c,* and *Utx*) and a histone methyltransferase (e.g., *Ehmt1*); and adapter molecules associated with the maintenance of higher-order chromosomal organization, including chromosomal ATPase (e.g., *Smc1a, Smc4,* and *Smc6*), high-mobility group (HMG)-box (e.g., *Hmg20b* and *Hmg20a*), and non-histone chromodomain (e.g., *Cbx1* and *Cbx4*) proteins. These observations suggest that REST and CoREST modulate transcription of this repertoire of epigenetic factors in a glial subtype and developmental stage specific manner. Indeed, recent studies show that epigenetic modulators, such as HDACs (class I, II, and IV), are differentially expressed during OL lineage elaboration [38], [69], [70] where they play important roles in regulating developmental stage specific stage gene expression. Furthermore, many of these factors are associated with REST, including members of the SWI/SNF factor family that serve as integral components of REST and CoREST repressor complexes [11], [15] and HMG-box proteins that differentially modulate REST target gene expression [71]. These findings suggest that REST and CoREST mediate the elaboration of distinct glial chromatin environments at both transcriptional and post-transcriptional levels.

#### Cell cycle regulation

REST and CoREST targeted genes encoding various cell cycle regulators (Table S6). These include Ccnd1, which modulates the G1/S phase transition [72] and Rbl1, which enforces the G2/M phase checkpoint [73]. Interestingly, Rb1 promotes cell cycle exit, in part, by maintaining histone H3 lysine 27 (H3K27) trimethylation marks on cell cycle genes [74]. These examples illustrate how REST and CoREST chromatin-based regulatory mechanisms lead to glial subtype specification and OL lineage maturation [74].

### Association of Promoter Occupancy with Developmental Gene Expression Profiles

To assess the potential roles of REST and CoREST in the modulation of glial gene expression programs, we also correlated changing profiles of REST and CoREST promoter occupancy with differential target gene expression patterns in cell types associated with critical developmental transitions. We examined specification of ASs (NSCs→ASs) and OLpres (N/OPs→OLpres) and progressive stages of OL lineage maturation (OLpres→OLpros, OLpros→ pmOLs, and pmOLs→myOLs) (Table 1).

Specifically, we identified genes that displayed either gain or loss of REST or CoREST promoter occupancy and also exhibited differential expression between the two cell types comprising the developmental transition. Among these, we found subsets of genes with gain of REST or CoREST promoter occupancy during the transitions that were associated with both gene activation and repression. Conversely, we also identified subsets of genes with loss of REST or CoREST promoter occupancy during the transitions that were similarly associated with both gene activation and repression. For example, among genes where REST was not bound in pmOLs but bound in myOLs, 197 genes were up regulated and 83 were down regulated in myOLs. Similarly, among genes where CoREST was not bound in N/OPs but bound in OLpres, 299 genes were up regulated and 167 genes were down regulated in OLpres. In contrast, among genes where REST was bound in NSCs but not bound in ASs, 90 genes were up regulated and 270 genes were down regulated in ASs. Also, among genes where CoREST was bound in OLpros but not bound in pmOLs, 41 genes were up regulated and 127 genes were down regulated in pmOLs. These cumulative results suggest that differential combinations of REST and CoREST promoter occupancy states dynamically regulate the expression of a myriad of gene targets with important roles in glial lineage elaboration, including both positive (e.g., factors that regulate key glial transition states) and negative (e.g., factors that mediate alternate neural and non-neural lineages and processes) modulators of glial lineage maturation and homeostasis.

## Discussion

Although REST and CoREST are believed to orchestrate neurogenesis by regulating the expression of neuronal differentiation genes, our ChIP-chip studies suggest that these factors are also important for modulating NSC-mediated glial lineage specification and maturation. The developmental stage- and cell type-specific profiles of target genes that we uncovered include many factors known to promote AS and OL subtype specification and progressive stages of OL lineage maturation including myelination. We also identified target genes involved in a broad array of cell-intrinsic processes, cell-cell communications, and environmental signaling pathways that may similarly be involved in determining aspects of glial cell identity and function. Indeed, it is becoming increasingly clear that factors presumed to have more general or homeostatic functions, such as those with roles in endoplasmic reticulum (ER) stress, ubiquitin proteasome, and autophagy pathways, may be implicated in glial developmental biology and may be responsible for the pathogenesis of disorders with selective glial cell vulnerability, such as Pelizaeus-Merzbacher disease and Vanishing White Matter (VWM) disease [75]. In addition, through corresponding gene expression analyses, we observed that changes in REST and CoREST promoter occupancy are associated with complex profiles of gene regulation, including both activation and repression. These findings are consistent with the emerging view that REST and CoREST complexes act with high degrees of context-specificity depending on developmental stage, cell type, and target gene locus.

Our results strongly suggest that, in addition to modulating genes involved in neuronal differentiation, REST and CoREST also selectively regulate genes encoding factors that promote the acquisition of glial cell identity and function. For example, REST and CoREST targeted genes are involved in key developmental pathways that mediate AS specification, including Notch, JAK-STAT, BMP, FGF, and EGF signaling, and OL specification and progressive maturation, such as PDGF, SHH, MAPK, and FGF signaling (Table S1) [76]. In addition, genes targeted by REST and CoREST encode a number of transcription factors that, in various combinations, govern almost every aspect of OL lineage specification, proliferation, and terminal differentiation including myelination. These include the HLH (e.g., *E2A, HEB, Mash1, Olig2,* and *Id4*), Hox (e.g., *Hoxa2*), POU (e.g., *Brn2*), Sox (e.g., *Sox4, Sox8,* and *Sox11*), Nkx (e.g., *Nkx6.1*), and zinc finger (e.g., *Myt1* and *Zfp488*) transcription factor families [77]. REST and CoREST also targeted many genes that encode components of the myelin sheath (e.g., *Mal, Mobp,* and *Mpzl1*).

Our results strongly suggest that REST and CoREST also modulate the specific deployment of a very wide range of epigenetic factors that may play important roles in sculpting glial cell identity and function. These include genes encoding DNA methylation factors (e.g., *Dnmt1, Mbd2, Mbd3,* and *Mbd6*); SWI/SNF chromatin remodeling enzymes (e.g., *Smarcd2, Smarcb1, Smarcad1,* and *Smarca1*); histone deacetylases (e.g., *Hdac6* and *Hdac7a*); histone demethylases (e.g., *Utx* and *Smcx*), including Jumonji-enzymes (e.g., *Jarid1c, Jarid1d, Jmjd1a,* and *Jmjd4*); and other adapter molecules associated with both euchromatic (e.g., *Hmg20a*) and heterochromatic (e.g., *Cbx5* and *Hmg20b*) states that may activate glial lineage specific genes and repress genes that are expressed in alternate lineages. For example, methyl-CpG binding domain proteins (MBDs) are generally thought to be expressed only in neurons and to repress glial genes [78]. Intriguingly, we found that REST targeted *Mbd2* in OLpre, *Mbd6* in pmOL, and *Mbd3* in myOL, while CoREST targeted *Mbd6* in pmOL and myOL and *Mbd3* in myOL. These MBD genes contain RE1s, and our observations further suggest that REST and CoREST selectively modulate the expression of these genes during progressive stages of OL lineage maturation. In addition, the cell- and tissue-specific expression profiles and functions of various HDAC enzymes are believed to be important for many different aspects of neural lineage specification, maturation, and developmental plasticity [79], [80]. However, the mechanisms governing HDAC expression are not well characterized. We found that during OL lineage elaboration, REST and CoREST targeted genes encoding HDACs from class II (e.g., *Hdac6* and *Hdac7*) and class III (e.g., *Sirt1, Sirt2,* and *Sirt5*). Together, these observations suggest that REST and CoREST orchestrate a spectrum of developmentally regulated and highly environmentally responsive epigenetic processes that differentially control seminal glial fate decisions.

Furthermore, our results strongly suggest that REST and CoREST also regulate various aspects of the microtubule network and cytoskeletal dynamics that are critical for OL maturation and myelination. Interestingly, in the OL lineage, Sirt2 is co-expressed with cyclic nucleotide phosphodiesterase (CNP), an OL marker, and has been implicated in the regulation of OL maturation and myelination, in part, because it can act as a microtubule tubulin deacetylase [81], [82]. Sirt2 has also been noted to interact with HDAC6, which can similarly act as a microtubule tubulin deacetylase [81], [82]. Unlike previous studies, we found that HDAC6 is highly expressed in myOLs suggesting that it may function in concert with Sirt2 and may be important for ensuring the fidelity of OL lineage progression. In addition, REST and CoREST targeted various genes encoding factors involved in microtubule growth, stability, and function including members of the tubulin (e.g., *Tubb4, Tubb2a, Tuba2, Tubd1,* and *Tubg1*), tubulin chaperone (e.g., *Tbca*), tubulin tyrosine ligase-like (e.g., *Ttll1* and *Ttll4*), microtubule associated protein (e.g., Mapt), stathmin (e.g., *Stmn1, Stmn2, Stmn3,* and *Stmn4*), kinesin (e.g., *Kif9, Kif6, Kif3c, Kif2a,* and *Kif22*), and dynein (e.g., *Dync2li1, Dync2h1, Dync1li1, Dync1i2, Dync1h1,* and *Dnaic1*) families.

Moreover, our results also suggest that, in glial cells, REST and CoREST modulate genes encoding various members of the nuclear receptor superfamily (e.g., *Nr5a1, Nr4a3, Nr3c1, Nr2f2, Nr2e3, Nr2e1, Nr1h4,* and *Nr1d2*), which act as dynamic sensors for the extracellular and intracellular milieu and have crucial roles in controlling neural development, homeostasis, and environmental responses. Intriguingly, a recent study reported that a different nuclear receptor, Nr4a2 (Nurr1), promotes a neuroprotective anti-inflammatory response in both microglia and ASs through CoREST-mediated transrepression of NF-kB activated pro-inflammatory genes and further suggested that a similar CoREST-mediated pathway may be widely used by other nuclear receptors, particularly members of the Nr4a family [83]. While we did not find that CoREST targets *Nr4a2* in ASs, our observations raise the interesting possibility that members of the nuclear receptor superfamily may act through REST- and/or CoREST-mediated transactivation and/or transrepression of target genes while also being subject to context-specific transcriptional regulation by REST and CoREST.

Indeed, this conclusion is supported by our observations that the *Nr2e1 (Tlx)* gene is targeted by both REST and CoREST, along with other studies showing that the Nr2e1 protein can interact directly with the histone demethylase, LSD1, a member of the CoREST complex, and recruit it to genomic Nr2e1 binding sites where it remodels the local chromatin environment [84]. In addition, further layers of complexity in REST-CoREST-nuclear receptor regulation and function are suggested by the recent finding that Nr2e1 participates in a dual negative transcriptional feedback loop with the microRNA, *miR-9* [85], which also has similar transcriptional regulatory relationships with REST and CoREST [16], [86].

This example illustrates how REST and CoREST seem to be at the nexus of the intricate circuitry embedded within epigenetic networks, intracellular signaling pathways, metabolic processes, and developmental programs responsible for encoding cell identity and function within the nervous system. Our results further imply that many families of genes not previously implicated in glial cell biology are modulated by REST and CoREST in a developmental stage- and cell type specific manner, which suggests that they may have selective roles in promoting aspects of glial cell identity, function, and connectivity and/or repressing characteristics of alternate lineages. These include but are not limited to genes encoding factors that define cell surface identity and connectivity (e.g., olfactory receptors, vomeronasal receptors, and transmembrane proteins) and promote homeostasis (e.g., solute carriers, DNA repair enzymes, and ER stress, cell cycle regulatory, ubiquitin-proteasome, and apoptosis-associated factors) as well as many additional families of proteins. Further characterization of these context-specific REST and CoREST transcriptional submodules and their regulatory topologies is necessary not only for understanding the control of glial lineage elaboration but also for defining the emergent properties of REST and CoREST networks and their dynamic functions, including potential roles in disease pathogenesis.

For example, REST inactivation, over expression, and copy number variation are observed in various cancer phenotypes, where they may promote loss of cellular identity and transformation through alterations in genomic stability, DNA methylation, chromatin remodeling, and deregulation of oncogenes and tumor suppressor genes. Indeed, REST, itself, is thought to have paradoxical functions as both a tumor suppressor gene and an oncogene, depending on the cellular context. Our results suggest that, in glial cells, both REST and CoREST modulate genes in key pathways responsible for the formation of glial tumors, such as glioblastoma multiforme (GBM). These include but are not limited to p53 (e.g., *Mdm4* and *Cdkn2a*), retinoblastoma (e.g., *Rb1*), growth factor (e.g., *Pdgfrb, Fgfr1, Bambi,* and *Smad4*), and receptor tyrosine kinase phosphatidylinositol-3-OH kinase (RTK-PI3K) (e.g., *Met, NF1,* and *Akt1*) signaling pathways. Because CoREST may regulate these important pathways, exhibits close functional relationships with REST, and has the capacity to recruit factors known to modulate cellular transformation such as the transcriptional corepressor, CtBP [87], and the oncogene, Znf217 [88], [89], [90], our data supports the conclusion that CoREST, like REST, may also play a key role in cellular transformation in the nervous system.

Furthermore, REST and CoREST also targeted genes associated with oligodendrogliopathies. For example, we found genes from genomic loci linked to multiple sclerosis (e.g., *Abca8a, Il2ra, Irf1, Prkca, Slc1a3, Tap2,* and *Ttyh2*), the most common cause of central nervous system demyelinating disease, and multisystem atrophy (e.g., *Tppp*), a primarily glial disorder that is related to Parkinson's disease and characterized by deregulation in the metabolism of myelin basic protein and tubulin polymerization promoting protein [91]. These observations imply that, because of their broad range of effects on DNA methylation, chromatin remodeling, and gene modulation, REST and CoREST may be key targets for epigenetic reprogramming not only in cancer but also in many other glial diseases, particularly as epigenetic remodeling is known to be important for modulating progressive stages of OL lineage maturation [69], [92], [93], [94].

In this study, we suggest that REST and CoREST each play essential, distinctive and interrelated roles in promoting the fidelity of glial cell identity, lineage maturation, maintenance of homeostasis, and neural plasticity. REST and CoREST are known to serve as nodes in highly integrated epigenetic regulatory networks that include ncRNAs with multiple layers of bidirectional feedback controls [16]. Therefore, our observations imply that these REST-CoREST-ncRNA epigenetic networks and the genes that they influence may orchestrate the developmental coupling of neurogenesis and gliogenesis, cellular migration, and neural network integration and plasticity by modulating AS functions, including regulation of microenvironmental niches and synaptic plasticity, as well as OL functions, including mediation of neuronal-glial interactions and axonal myelination. Furthermore, these complex developmental relationships may be critical for better characterizing a wide range of central nervous system pathologies and for developing novel diagnostic and therapeutic strategies that involve dynamic epigenetic reprogramming of REST- and CoREST-mediated neural fate decisions.

## Materials and Methods

### Cell Cultures

Culture preparations were generated as previously described with minor modifications [49], [95], [96], [97], [98], [99], [100]. Briefly, multilineage potential and more lineage restricted progenitor species derived from embryonic day 14.5 (E14.5) ventral forebrain regions of CD1 mice were plated and propagated in serum free media (SFM) containing specified factors for various time intervals, and subsequently examined by immunofluorescence microscopy to define developmental neural lineage profiles [49], [95], [96], [97], [98], [99], [100], by Western blot analysis to detect REST and CoREST protein expression [95], [101], and by Qchip and ChIP-chip to identify DNA binding sites for REST and CoREST as previously described [102], [103], [104]. Primary neural stem cell (NSC) clones were generated by application of basic fibroblast growth factor (bFGF, 10 ng/ml) for 7 days in vitro (DIV) and then dissociated using 0.05% trypsin (GIBCO) for 15 minutes at 37°C. Individual cells were re-propagated in bFGF for an additional 2 DIV to form secondary NSC clones that were used for experiments and are referred to herein as NSCs. The expansion of secondary NSC clones was limited to 2 DIV to avoid inclusion of differentiated neural species from this culture condition. This culture paradigm eliminates intermediate neural progenitor species and other proliferating neural developmental cell types present in primary NSC clones and yields >98% homogeneity. The number of DIV required for the elaboration of all differentiated progeny was determined to satisfy several essential developmental and cellular criteria, including homogeneity of the developmental lineage species examined (>95%) and the absence of alternative neural species. Lineage-restricted neuronal-OL progenitor (N/OP) clones [49] were generated from dissociated primary NSC clones by application of bFGF and the N-terminal active form of Shh (N-Shh,100 ng/ml) for 2 DIV [98] to achieve >98% homogeneity. OL precursor cells (OLpres) comprised >98% of the cell population that was generated from N/OP clones by application of platelet-derived growth factor (PDGF-AA, 10 ng/ml) after 2 DIV. Cellular species representing progressive stages of OL lineage maturation were generated by propagation in SFM containing laminin (3 µg/ml, BD Biosciences) on poly-D-lysine (PDL) coated culture dishes at non-confluent densities. OL progenitors (OLpros) were generated from OL precursor species by application of PDGF-AA (10 ng/ml) for an additional 2–4 DIV to achieve >95% homogeneity. Cell culture composition and homogeneity remained similar for the two subsequent developmental stages of OL lineage maturation. Post-mitotic OLs (pmOLs) and mature, myelin expressing OLs (myOLs) were generated from OL progenitors by withdrawal of PDGF-AA for an additional 2 and 4 DIV, respectively [105]. Astrocytes (ASs), comprising >98% purity, were generated by dissociation of secondary NSC clones using 0.05% trypsin for 15 minutes at 37°C and re-propagation of individual cells in epidermal growth factor (EGF) for 7 DIV with subsequent addition of bone morphogenetic protein 2 (BMP2) for 5 DIV [99], [100].

GABAergic neurons (GABANs) were generated by application of BMP2 (10 ng/ml) to N/OPs propagated on poly-D-lysine (PDL) coated culture dishes with the addition of laminin (3 µg/ml, BD Biosciences) for 2 DIV [98]. Cholinergic neurons (CHOLNs) were generated from E14.5 ventral forebrain-derived NSCs by minor modification of a previously described method [106]. Individual cells from primary NSC clones were plated in Neurobasal medium (GIBCO) supplemented with N2 on PDL coated culture dishes with the addition of laminin (3 µg/ml), bFGF (10 ng/ml), N-Shh (100 ng/ml) and nerve growth factor (NGF, 200 ng/ml) for 2 DIV followed by application of N-Shh and NGF for an additional 14 DIV. Medium spiny projection neurons (MSNs) were generated from E14.5 ventral forebrain derived NSCs by the modification of a previously defined method [107]. Individual cells from primary NSC clones were plated on PDL-coated cultures dishes containing Neurobasal, B27 and laminin (3 µg/ml) and were treated with bFGF (10 ng/ml), N-Shh (50 ng/ml) and brain derived neurotrophic factor (BDNF, 50 ng/ml) for 1 DIV followed by application of BDNF (100 ng/ml) for another 14 DIV. Glutamatergic neurons (GLUTNs) were generated directly from radial glial cells (RG) that were obtained from dorsal forebrain derived primary NSC clones. Briefly, individual cells dissociated from dorsal forebrain derived NSC clones were propagated in SFM on PDL-coated dishes in SFM supplemented with laminin (3 µg/ml) in the presence of bFGF (10 ng/ml) and LIF (10 ng/ml) for 2 DIV to elaborate RG of >97% homogeneity. GLUTN were subsequently generated by withdrawal of bFGF and LIF from RG for an additional 4 DIV. To ensure that all neuronal subtypes species reached >99% homogeneity before being utilized in ChIP experiments, we added 10 µm cytosine arabinoside (Sigma) to the culture media 24 hours before harvesting the cells and thereby eliminated all dividing cells by apoptosis [108], [109], [110]. We followed institutional IACUC guidelines for experiments in which primary mouse tissue specimens were utilized.

### Cell Line Cultures

The mouse oligodendrocyte precursor cell line, Oli Neu [111] was a kind gift of Dr. J. Trotter (University of Mainz, Germany). Cells were grown on PDL-coated culture dishes. Immature Oli-neu cells were maintained in growth medium consisting of DMEM supplemented with 2 mM L-glutamine, 1 mM sodium pyruvate, 10 ng/ml biotin, 100 mg/ml apotransferrin, 100 mM putrescine, 20 nM progesterone, 30 nM sodium selenite, 5 mg/ml insulin and 1% horse serum. The induction of oligodendrocyte differentiation was promoted by switching cells to media containing 1 mM dibutryl-cAMP (Sigma) as previously described [111]. Oli-neu cells were continuously maintained at 37°C in 5% CO2 at a humid atmosphere.

### Immunofluorescence Microscopic Analysis

Immunofluorescence microscopic analysis was performed as we have previously described [95], [96].

### Specific Antibody Preparations

All antibodies exhibited selective immunoreactivity for mouse cells and tissue sections, and each antibody exhibited a complete absence of alternate cross-reactivity. The following antibodies were utilized: CoREST, REST, and normal rabbit IgG (1∶100, Upstate, Temecula, CA, USA), the neuroepithelial marker (nestin, mIgG1, 1∶200, Pharmingen), N/OP markers (Olig2, goat IgG, 1∶300, R&D and Mash1, mIgG1, 1∶100, Pharmingen), OLpre marker (NG2, rIg, 1∶500, Chemicon), the OLpro marker (O4, mIgM, 1∶700, Sigma), the pmOL markers (GC/O1, mIgM, 1∶350, Chemicon), the myOL marker (MBP, mIgG2b, 1∶500 Sternberger Monoclonals) and the AS marker (GFAP, mIgG1, 1∶400, Sigma). Isotype specific secondary antibodies were utilized at a 1∶1500 dilution according to the required fluorophore combinations (Invitrogen). Secondary antibodies utilized for Western blot analysis were HRP conjugated (GE Healthcare).

### Western Blot Analysis

Cell cultures were processed for Western blot analysis as described previously [95], [101], [112], [113]. Briefly, cells were homogenized in nine volumes of buffer comprising 0.32 m sucrose, 50 mm Tris-HCl, pH 8.0, EDTA-free protease inhibitors cocktail (Roche) and 0.5 mm phenylmethylsulfonyl fluoride using a glass–Teflon homogenizer (10 strokes at 800 rpm) on ice, centrifuged at 900 g for 10 min and lysed in sodium dodecyl sulfate sample-loading buffer for Western blot analysis.

### Growth Factor Preparations

To generate the various neural stem, progenitor and more differentiated glial species the following growth factor preparations were utilized: recombinant bFGF (Collaborative Biomedical Products), recombinant EGF, N-Shh and PDGF-AA (R&D Systems) and BMP2 (gift from Genetics Institute). To generate the comparative neuronal subtype species the following additional growth factor preparations were utilized: recombinant mouse β-NGF (R&D Systems), human BDNF (BioVision), recombinant BMP2 (gift from Genetics Institute) and recombinant LIF (Chemicon).

### Quantitative Chromatin Immunoprecipitation (QChIP)

Cultures were prepared essentially as previously described [49], [98] and used in chromatin immunoprecipitation (ChIP) [102], [103], [104], with minor modifications. Between 1×10^6^ and 3×10^6^ cells were used per antibody. The following antibodies were utilized: CoREST, REST, and normal rabbit IgG (Upstate, Temecula, CA, USA). An additional control included the absence of antibody (input chromatin). Antibodies were first validated using a peptide competition assay. For 1×10^6^ cells, 10 µg of antibody was used. Enrichment of fragments by ChIP was quantified by using 1 µl of ChIP product for real-time PCR using the SYBR® Green kit (Applied Biosystems, Foster City, CA, USA) in a 7000 Real Time PCR system® (Applied Biosystems, CA, USA). Validated CoREST and REST promoter binding sites were used as positive controls, while a non-target gene promoter was used as a negative control. ΔC_T_ values were obtained using the ΔΔC_T_ method [114]. For validation of ChIP-chip results, primers, 50 to 150 base pairs in length, were designed to flank the binding peak within each of the respective promoter sequences.

### ChIP-Chip Assays

To investigate the specific roles of CoREST and REST throughout early neural developmental stages, we examined the differential binding profiles of CoREST and REST by determining their gene-specific targets through a series of ChIP-chip experiments. Chromatin immunoprecipitation is an *in vivo* technique that can be used to identify transcription factor binding sites. To determine whether the CoREST and REST antibodies were specific for known targets, ChIP was first performed in an Oli-Neu cell line [111] with a CoREST antibody, REST antibody, control IgG, or no antibody (input). Samples were then analyzed by quantitative PCR (QPCR) using primers specific for both a known and previously validated CoREST and REST target gene, GluR2, and a negative control [4], [115]. Chromatin immunoprecipitation targets were determined by utilizing a fold-enrichment greater than 1.5. We then performed a series of ChIP-chip experiments in order to build a comprehensive profile of CoREST and REST target genes throughout early neural developmental stages. The mouse promoter array was based on MM8/mouse genome Build 36 from February 2006 (NimbleGen Systems, Madison, WI). The design is based only on normal RefSeq genes (17,355 genes) and includes 2,000 base pairs upstream of the transcriptional start sites and 500 base pairs downstream. The probe size ranges from 50-75 base pairs and the spacing interval is 100 base pairs.

### ChIP-Chip Data Analysis

Analysis was performed essentially as previously described [116]. Enrichment was calculated for each probe by computing the log-ratio value for the ChIP immunoprecipitation product in comparison to the input chromatin. For all ChIP-chip experiments, in order to find promoter peaks, a maximum log-ratio value for a window consisting of three consecutive probes was determined for both experimental data and a random permutation of the data. A positive threshold was then established to determine the probability for real enrichment. This positive threshold was determined by examining signals of known CoREST and REST binding sites, GluR2, and calbindin, respectively [4], [115]. A 90% positive threshold was used. The gene target lists were generated based on the intersection of genes across a minimum of two arrays for each experimental paradigm. We validated these ChIP-chip results with QchIP in a representative sample of cell types from our developmental paradigm and found 83% and 94% correlation for CoREST and REST, respectively. These results indicate that the ChIP-chip technique and data analysis methods used to characterize CoREST and REST target genes are effective approaches for identifying valid binding sites.

### Functional Classification of Target Genes

Target genes were functionally annotated by the use of Ingenuity Pathways Analysis (Ingenuity® Systems, www.ingenuity.com).

### Gene Expression Assays and Analysis

The gene expression array was based on MM8/mouse genome build 36 from February 2006 (NimbleGen, Madison, WI). The Robust Multi-Array Average (RMA) algorithm was used and data was also analyzed by the significant analysis of microarray methods (SAM). Each of the different cell types was compared to neural stem cells. Three biological replicates were conducted for each cell type.

## Supporting Information

Table S1 Composite profiles of REST and CoREST target genes in glial developmental cell types. For the ChIP-chip data, the value 1 denotes gene promoter occupancy. For the gene expression data, the value represents the expression in the cell type relative to expression in neural stem cells. The Robust Multi-Array Average (RMA) algorithm and significant analysis of microarray methods (SAM) were used to analyze the expression data. Three biological replicates were conducted for each cell type.
(2.14 MB XLS)

Table S2 Comparative analysis of pathways enriched in composite profiles of REST, CoREST, and REST-CoREST target genes. The values in each row represent the degree of pathway enrichment (−LogP) for each set of target genes. Only pathways exhibiting high degrees of statistical significance (−LogP>2) are included. Target genes were analyzed using Ingenuity Pathways Analysis (Ingenuity® Systems, www.ingenuity.com).
(0.03 MB XLS)

Table S3 Comparative analysis of pathways enriched in REST and CoREST target genes for individual glial developmental cell types. The values in each row represent the degree of pathway enrichment (−LogP) for each set of target genes. Only pathways exhibiting statistical significance (−LogP>1.3) are included. Target genes were analyzed using Ingenuity Pathways Analysis (Ingenuity® Systems, www.ingenuity.com).
(0.03 MB XLS)

Table S4 Selective profiles of REST and CoREST target genes encoding factors with known roles in glial developmental cell types.
(0.76 MB XLS)

Table S5 Selective profiles of REST and CoREST target genes encoding epigenetic factors in glial developmental cell types.
(0.12 MB DOC)

Table S6 Selective profiles of REST and CoREST target genes encoding cell cycle regulators in glial developmental cell types.
(0.18 MB DOC)
