# Supplementary material for: Differential Deployment of REST and CoREST Promotes Glial Subtype Specification and Oligodendrocyte Lineage Maturation
Source: PLoS One. 2009 Nov 3;4(11):e7665. doi: 10.1371/journal.pone.0007665 (PMC2766030; doi:10.1371/journal.pone.0007665)
Supplement: Table S5 — Selective profiles of REST and CoREST target genes encoding epigenetic factors in glial developmental cell types. (0.12 MB DOC) [file pone.0007665.s005.doc]

|  | **REST** | | | | | | **CoREST** | | | | | |
| --- | --- | --- | --- | --- | --- | --- | --- | --- | --- | --- | --- | --- |
| **Gene** | **OLpres** | **OLpros** | **pmOLs** | **myOL s** | **ASs** | **REST** | **OLpres** | **OLpros** | **pmOLs** | **myOLs** | **ASs** | **CoREST** |
| Hist1h4a | 0 | 0 | 1 | 1 | 0 | **2** | 0 | 0 | 1 | 1 | 0 | **2** |
| Scmh1 | 0 | 0 | 1 | 1 | 0 | **2** | 0 | 0 | 1 | 1 | 0 | **2** |
| Smc4l1 | 0 | 0 | 1 | 1 | 0 | **2** | 1 | 0 | 0 | 1 | 0 | **2** |
| Mbd6 | 0 | 0 | 1 | 0 | 0 | **1** | 0 | 0 | 1 | 1 | 0 | **2** |
| Smarca2 | 0 | 0 | 0 | 1 | 0 | **1** | 0 | 0 | 1 | 1 | 0 | **2** |
| Smc1l1 | 0 | 0 | 1 | 0 | 0 | **1** | 0 | 0 | 1 | 1 | 0 | **2** |
| Smc6l1 | 0 | 1 | 0 | 0 | 0 | **1** | 1 | 1 | 0 | 0 | 0 | **2** |
| Hmg20b | 0 | 0 | 0 | 0 | 0 | **0** | 0 | 0 | 1 | 1 | 0 | **2** |
| Sin3b | 0 | 0 | 0 | 0 | 0 | **0** | 0 | 0 | 1 | 1 | 0 | **2** |
| Hist1h2af | 0 | 1 | 1 | 0 | 1 | **3** | 0 | 0 | 1 | 0 | 0 | **1** |
| Smarcd2 | 0 | 0 | 1 | 1 | 0 | **2** | 0 | 0 | 0 | 1 | 0 | **1** |
| Suv39h2 | 0 | 0 | 1 | 1 | 0 | **2** | 0 | 0 | 1 | 0 | 0 | **1** |
| Cbx4 | 0 | 0 | 1 | 0 | 0 | **1** | 0 | 0 | 1 | 0 | 0 | **1** |
| Chmp5 | 0 | 0 | 1 | 0 | 0 | **1** | 0 | 0 | 1 | 0 | 0 | **1** |
| Hdac7a | 0 | 1 | 0 | 0 | 0 | **1** | 0 | 0 | 1 | 0 | 0 | **1** |
| Mbd3 | 0 | 0 | 0 | 1 | 0 | **1** | 0 | 0 | 0 | 1 | 0 | **1** |
| Utx | 0 | 1 | 0 | 0 | 0 | **1** | 0 | 0 | 1 | 0 | 0 | **1** |
| Cbx5 | 0 | 0 | 0 | 0 | 0 | **0** | 0 | 0 | 0 | 0 | 1 | **1** |
| Chaf1b | 0 | 0 | 0 | 0 | 0 | **0** | 0 | 0 | 0 | 1 | 0 | **1** |
| Chd1 | 0 | 0 | 0 | 0 | 0 | **0** | 0 | 1 | 0 | 0 | 0 | **1** |
| Hmg20a | 0 | 0 | 0 | 0 | 0 | **0** | 1 | 0 | 0 | 0 | 0 | **1** |
| Hp1bp3 | 0 | 0 | 0 | 0 | 0 | **0** | 0 | 1 | 0 | 0 | 0 | **1** |
| Jarid1d | 0 | 0 | 0 | 0 | 0 | **0** | 0 | 1 | 0 | 0 | 0 | **1** |
| Pcgf2 | 0 | 0 | 0 | 0 | 0 | **0** | 0 | 0 | 1 | 0 | 0 | **1** |
| Rhox2 | 0 | 0 | 0 | 0 | 0 | **0** | 1 | 0 | 0 | 0 | 0 | **1** |
| Rhox9 | 0 | 0 | 0 | 0 | 0 | **0** | 0 | 0 | 1 | 0 | 0 | **1** |
| Smarca1 | 0 | 0 | 0 | 0 | 0 | **0** | 0 | 1 | 0 | 0 | 0 | **1** |
| Smarca3 | 0 | 0 | 0 | 0 | 0 | **0** | 1 | 0 | 0 | 0 | 0 | **1** |
| Smarcad1 | 0 | 0 | 0 | 0 | 0 | **0** | 0 | 1 | 0 | 0 | 0 | **1** |
| Smarcal1 | 0 | 0 | 0 | 0 | 0 | **0** | 0 | 0 | 0 | 1 | 0 | **1** |
| Smarcb1 | 0 | 0 | 0 | 0 | 0 | **0** | 0 | 0 | 1 | 0 | 0 | **1** |
| Ehmt1 | 0 | 1 | 1 | 1 | 0 | **3** | 0 | 0 | 0 | 0 | 0 | **0** |
| Jmjd1a | 0 | 1 | 1 | 1 | 0 | **3** | 0 | 0 | 0 | 0 | 0 | **0** |
| Ash2l | 0 | 1 | 0 | 0 | 0 | **1** | 0 | 0 | 0 | 0 | 0 | **0** |
| Cbx8 | 0 | 0 | 0 | 1 | 0 | **1** | 0 | 0 | 0 | 0 | 0 | **0** |
| Chrac1 | 0 | 0 | 0 | 1 | 0 | **1** | 0 | 0 | 0 | 0 | 0 | **0** |
| Dnmt1 | 0 | 0 | 1 | 0 | 0 | **1** | 0 | 0 | 0 | 0 | 0 | **0** |
| Hdac6 | 0 | 0 | 1 | 0 | 0 | **1** | 0 | 0 | 0 | 0 | 0 | **0** |
| Hist1h2bm | 0 | 0 | 1 | 0 | 0 | **1** | 0 | 0 | 0 | 0 | 0 | **0** |
| Hist1h3e | 0 | 0 | 1 | 0 | 0 | **1** | 0 | 0 | 0 | 0 | 0 | **0** |
| Hist1h4d | 0 | 1 | 0 | 0 | 0 | **1** | 0 | 0 | 0 | 0 | 0 | **0** |
| Hist1h4f | 0 | 0 | 1 | 0 | 0 | **1** | 0 | 0 | 0 | 0 | 0 | **0** |
| Hist2h2aa2 | 0 | 0 | 0 | 0 | 1 | **1** | 0 | 0 | 0 | 0 | 0 | **0** |
| Hist2h3c1 | 0 | 0 | 0 | 0 | 1 | **1** | 0 | 0 | 0 | 0 | 0 | **0** |
| Hr | 0 | 0 | 1 | 0 | 0 | **1** | 0 | 0 | 0 | 0 | 0 | **0** |
| Hspbap1 | 0 | 0 | 1 | 0 | 0 | **1** | 0 | 0 | 0 | 0 | 0 | **0** |
| Jarid1c | 0 | 0 | 1 | 0 | 0 | **1** | 0 | 0 | 0 | 0 | 0 | **0** |
| Mbd2 | 1 | 0 | 0 | 0 | 0 | **1** | 0 | 0 | 0 | 0 | 0 | **0** |
| Mizf | 0 | 0 | 1 | 0 | 0 | **1** | 0 | 0 | 0 | 0 | 0 | **0** |
| Pcgf3 | 0 | 0 | 1 | 0 | 0 | **1** | 0 | 0 | 0 | 0 | 0 | **0** |
| Smarcc1 | 0 | 0 | 1 | 0 | 0 | **1** | 0 | 0 | 0 | 0 | 0 | **0** |
