# Supplementary material for: Differential Deployment of REST and CoREST Promotes Glial Subtype Specification and Oligodendrocyte Lineage Maturation
Source: PLoS One. 2009 Nov 3;4(11):e7665. doi: 10.1371/journal.pone.0007665 (PMC2766030; doi:10.1371/journal.pone.0007665)
Supplement: Table S6 — Selective profiles of REST and CoREST target genes encoding cell cycle regulators in glial developmental cell types. (0.18 MB DOC) [file pone.0007665.s006.doc]

|  | **REST** | | | | | | **CoREST** | | | | | |
| --- | --- | --- | --- | --- | --- | --- | --- | --- | --- | --- | --- | --- |
| **Gene** | **OLpre s** | **OLpro s** | **pmOL s** | **myOL s** | **ASs** | **REST** | **OLpre s** | **OLpro s** | **pmOL s** | **myOL s** | **ASs** | **CoREST** |
| Nek8 | 0 | 0 | 1 | 1 | 0 | **2** | 0 | 0 | 1 | 1 | 0 | **2** |
| 2810452K22Rik | 0 | 0 | 0 | 1 | 0 | **1** | 0 | 0 | 1 | 1 | 0 | **2** |
| AI842396 | 0 | 0 | 0 | 1 | 0 | **1** | 0 | 0 | 1 | 1 | 0 | **2** |
| Ccnj | 0 | 0 | 0 | 0 | 1 | **1** | 0 | 1 | 0 | 0 | 1 | **2** |
| Mycbp | 0 | 0 | 0 | 1 | 0 | **1** | 0 | 0 | 1 | 1 | 0 | **2** |
| Pak1ip1 | 0 | 0 | 0 | 1 | 0 | **1** | 0 | 0 | 1 | 1 | 0 | **2** |
| Rasa4 | 0 | 0 | 0 | 1 | 0 | **1** | 0 | 0 | 1 | 1 | 0 | **2** |
| Vav3 | 0 | 1 | 0 | 0 | 0 | **1** | 1 | 1 | 0 | 0 | 0 | **2** |
| 6-Sep | 0 | 0 | 0 | 0 | 0 | **0** | 0 | 0 | 1 | 1 | 0 | **2** |
| Arg1 | 0 | 0 | 0 | 0 | 0 | **0** | 1 | 0 | 0 | 1 | 0 | **2** |
| Cdk5rap2 | 0 | 0 | 0 | 0 | 0 | **0** | 1 | 0 | 0 | 0 | 1 | **2** |
| Mxd1 | 0 | 0 | 1 | 0 | 1 | **2** | 0 | 0 | 0 | 0 | 1 | **1** |
| 9130211I03Rik | 0 | 0 | 0 | 1 | 0 | **1** | 1 | 0 | 0 | 0 | 0 | **1** |
| Anapc10 | 0 | 0 | 1 | 0 | 0 | **1** | 0 | 1 | 0 | 0 | 0 | **1** |
| Ccna2 | 0 | 1 | 0 | 0 | 0 | **1** | 0 | 1 | 0 | 0 | 0 | **1** |
| Ccnd1 | 0 | 0 | 0 | 1 | 0 | **1** | 0 | 0 | 0 | 1 | 0 | **1** |
| Cdc34 | 0 | 1 | 0 | 0 | 0 | **1** | 0 | 0 | 1 | 0 | 0 | **1** |
| Cdk5r1 | 0 | 0 | 0 | 1 | 0 | **1** | 0 | 0 | 0 | 1 | 0 | **1** |
| Cdkn2c | 0 | 0 | 1 | 0 | 0 | **1** | 0 | 0 | 0 | 1 | 0 | **1** |
| Cited1 | 0 | 0 | 1 | 0 | 0 | **1** | 0 | 0 | 0 | 1 | 0 | **1** |
| F13b | 0 | 0 | 0 | 0 | 1 | **1** | 1 | 0 | 0 | 0 | 0 | **1** |
| G0s2 | 0 | 0 | 0 | 1 | 0 | **1** | 0 | 0 | 0 | 1 | 0 | **1** |
| Gas2 | 0 | 1 | 0 | 0 | 0 | **1** | 0 | 1 | 0 | 0 | 0 | **1** |
| Gata6 | 0 | 0 | 1 | 0 | 0 | **1** | 0 | 0 | 1 | 0 | 0 | **1** |
| Gspt1 | 0 | 0 | 0 | 1 | 0 | **1** | 0 | 1 | 0 | 0 | 0 | **1** |
| Lats2 | 0 | 0 | 0 | 0 | 1 | **1** | 1 | 0 | 0 | 0 | 0 | **1** |
| Nsfl1c | 1 | 0 | 0 | 0 | 0 | **1** | 1 | 0 | 0 | 0 | 0 | **1** |
| Nucks1 | 0 | 1 | 0 | 0 | 0 | **1** | 1 | 0 | 0 | 0 | 0 | **1** |
| Pak1 | 0 | 0 | 1 | 0 | 0 | **1** | 0 | 0 | 1 | 0 | 0 | **1** |
| Rbbp6 | 0 | 1 | 0 | 0 | 0 | **1** | 1 | 0 | 0 | 0 | 0 | **1** |
| Rbbp6 | 0 | 0 | 0 | 1 | 0 | **1** | 0 | 0 | 0 | 1 | 0 | **1** |
| Rbl1 | 0 | 0 | 0 | 1 | 0 | **1** | 0 | 0 | 0 | 1 | 0 | **1** |
| Terf2ip | 0 | 1 | 0 | 0 | 0 | **1** | 0 | 0 | 1 | 0 | 0 | **1** |
| Tpd52l1 | 0 | 0 | 0 | 1 | 0 | **1** | 0 | 0 | 0 | 1 | 0 | **1** |
| 5-Sep | 0 | 0 | 0 | 0 | 0 | **0** | 0 | 0 | 1 | 0 | 0 | **1** |
| 7-Sep | 0 | 0 | 0 | 0 | 0 | **0** | 0 | 1 | 0 | 0 | 0 | **1** |
| Armet | 0 | 0 | 0 | 0 | 0 | **0** | 0 | 0 | 1 | 0 | 0 | **1** |
| BC046343 | 0 | 0 | 0 | 0 | 0 | **0** | 0 | 0 | 1 | 0 | 0 | **1** |
| Ccnb1 | 0 | 0 | 0 | 0 | 0 | **0** | 0 | 0 | 0 | 1 | 0 | **1** |
| Ccnb3 | 0 | 0 | 0 | 0 | 0 | **0** | 0 | 1 | 0 | 0 | 0 | **1** |
| Ccnk | 0 | 0 | 0 | 0 | 0 | **0** | 0 | 1 | 0 | 0 | 0 | **1** |
| Cdc16 | 0 | 0 | 0 | 0 | 0 | **0** | 1 | 0 | 0 | 0 | 0 | **1** |
| Cdc2a | 0 | 0 | 0 | 0 | 0 | **0** | 1 | 0 | 0 | 0 | 0 | **1** |
| Cdca5 | 0 | 0 | 0 | 0 | 0 | **0** | 0 | 0 | 1 | 0 | 0 | **1** |
| Cdkl1 | 0 | 0 | 0 | 0 | 0 | **0** | 1 | 0 | 0 | 0 | 0 | **1** |
| Cdkn2a | 0 | 0 | 0 | 0 | 0 | **0** | 0 | 1 | 0 | 0 | 0 | **1** |
| Cnnm4 | 0 | 0 | 0 | 0 | 0 | **0** | 0 | 0 | 0 | 1 | 0 | **1** |
| Elf3 | 0 | 0 | 0 | 0 | 0 | **0** | 0 | 0 | 1 | 0 | 0 | **1** |
| Gata1 | 0 | 0 | 0 | 0 | 0 | **0** | 0 | 0 | 0 | 1 | 0 | **1** |
| Mdm4 | 0 | 0 | 0 | 0 | 0 | **0** | 1 | 0 | 0 | 0 | 0 | **1** |
| Plxdc1 | 0 | 0 | 0 | 0 | 0 | **0** | 0 | 0 | 1 | 0 | 0 | **1** |
| Rap2b | 0 | 0 | 0 | 0 | 0 | **0** | 0 | 1 | 0 | 0 | 0 | **1** |
| Serinc3 | 0 | 0 | 0 | 0 | 0 | **0** | 0 | 0 | 1 | 0 | 0 | **1** |
| Terf1 | 0 | 0 | 0 | 0 | 0 | **0** | 0 | 0 | 0 | 1 | 0 | **1** |
| Tert | 0 | 0 | 0 | 0 | 0 | **0** | 1 | 0 | 0 | 0 | 0 | **1** |
| Tssc4 | 0 | 0 | 0 | 0 | 0 | **0** | 0 | 0 | 1 | 0 | 0 | **1** |
| Tusc4 | 0 | 0 | 0 | 0 | 0 | **0** | 0 | 0 | 0 | 1 | 0 | **1** |
| Vav2 | 0 | 0 | 0 | 0 | 0 | **0** | 0 | 0 | 0 | 1 | 0 | **1** |
| 1110049F12Rik | 0 | 0 | 1 | 0 | 0 | **1** | 0 | 0 | 0 | 0 | 0 | **0** |
| AI317223 | 0 | 1 | 0 | 0 | 0 | **1** | 0 | 0 | 0 | 0 | 0 | **0** |
| Bcor | 0 | 0 | 0 | 1 | 0 | **1** | 0 | 0 | 0 | 0 | 0 | **0** |
| Bcorl1 | 0 | 0 | 1 | 0 | 0 | **1** | 0 | 0 | 0 | 0 | 0 | **0** |
| Brap | 0 | 0 | 0 | 1 | 0 | **1** | 0 | 0 | 0 | 0 | 0 | **0** |
| Cacybp | 0 | 0 | 1 | 0 | 0 | **1** | 0 | 0 | 0 | 0 | 0 | **0** |
| Ccndbp1 | 0 | 0 | 1 | 0 | 0 | **1** | 0 | 0 | 0 | 0 | 0 | **0** |
| Ccni | 0 | 0 | 1 | 0 | 0 | **1** | 0 | 0 | 0 | 0 | 0 | **0** |
| Ccnt2 | 0 | 0 | 1 | 0 | 0 | **1** | 0 | 0 | 0 | 0 | 0 | **0** |
| Cdc7 | 0 | 0 | 1 | 0 | 0 | **1** | 0 | 0 | 0 | 0 | 0 | **0** |
| Cdk5r2 | 0 | 0 | 1 | 0 | 0 | **1** | 0 | 0 | 0 | 0 | 0 | **0** |
| Cdkal1 | 0 | 1 | 0 | 0 | 0 | **1** | 0 | 0 | 0 | 0 | 0 | **0** |
| Cnnm1 | 0 | 0 | 0 | 1 | 0 | **1** | 0 | 0 | 0 | 0 | 0 | **0** |
| Dmtf1 | 0 | 0 | 0 | 1 | 0 | **1** | 0 | 0 | 0 | 0 | 0 | **0** |
| Gadd45g | 0 | 0 | 1 | 0 | 0 | **1** | 0 | 0 | 0 | 0 | 0 | **0** |
| Mtag2 | 0 | 0 | 0 | 1 | 0 | **1** | 0 | 0 | 0 | 0 | 0 | **0** |
| Mtbp | 1 | 0 | 0 | 0 | 0 | **1** | 0 | 0 | 0 | 0 | 0 | **0** |
| Nbl1 | 0 | 1 | 0 | 0 | 0 | **1** | 0 | 0 | 0 | 0 | 0 | **0** |
| Oit1 | 0 | 0 | 1 | 0 | 0 | **1** | 0 | 0 | 0 | 0 | 0 | **0** |
| Pdrg1 | 0 | 0 | 0 | 0 | 1 | **1** | 0 | 0 | 0 | 0 | 0 | **0** |
| Rac1 | 0 | 1 | 0 | 0 | 0 | **1** | 0 | 0 | 0 | 0 | 0 | **0** |
| Rap2a | 0 | 0 | 1 | 0 | 0 | **1** | 0 | 0 | 0 | 0 | 0 | **0** |
| Rb1 | 0 | 1 | 0 | 0 | 0 | **1** | 0 | 0 | 0 | 0 | 0 | **0** |
| Rbbp7 | 0 | 0 | 0 | 1 | 0 | **1** | 0 | 0 | 0 | 0 | 0 | **0** |
| Serinc1 | 0 | 0 | 1 | 0 | 0 | **1** | 0 | 0 | 0 | 0 | 0 | **0** |
| Tpt1 | 0 | 1 | 0 | 0 | 0 | **1** | 0 | 0 | 0 | 0 | 0 | **0** |
| Tusc1 | 0 | 0 | 0 | 1 | 0 | **1** | 0 | 0 | 0 | 0 | 0 | **0** |
| Wt1 | 0 | 0 | 1 | 0 | 0 | **1** | 0 | 0 | 0 | 0 | 0 | **0** |
